# A nationwide study of acquired C1-inhibitor deficiency in France: Characteristics and treatment responses in 92 patients

**DOI:** 10.1097/MD.0000000000004363

**Published:** 2016-08-19

**Authors:** Delphine Gobert, Romain Paule, Denise Ponard, Pierre Levy, Véronique Frémeaux-Bacchi, Laurence Bouillet, Isabelle Boccon-Gibod, Christian Drouet, Stéphane Gayet, David Launay, Ludovic Martin, Arsène Mekinian, Véronique Leblond, Olivier Fain

**Affiliations:** a Internal Medicine Department, Saint Antoine Hospital, Assistance Publique-Hôpitaux de Paris, DHU i2B, Paris 6 University, Paris; b Hematology Department, Pitié Salpétrière Hospital, Assistance Publique-Hôpitaux de Paris, Paris 6 University, Paris; c Immunology Laboratory, University Hospital, Grenoble; d Centre de Référence et d’Etude des Angioedèmes à Kinine (CREAK) , Grenoble; e Public Health Department, Tenon Hospital, Assistance Publique-Hôpitaux de Paris, Paris 6 University; f Immunology Laboratory, Georges Pompidou European Hospital, Assistance Publique-Hôpitaux de Paris, Paris 5 University, Paris; g Joint Unit 1036 CNRS-CEA-INSERM, University Grenoble Alpes; h Internal Medicine Department, University Hospital, Grenoble; i Université Joseph Fourier Grenoble, GREPI/AGIM CNRS FRE 3405, Grenoble; j Internal Medicine Department, La Conception Hospital, AP-HM, Marseille; k Internal Medicine and Clinical Immunology Department, Lille University Hospital; l LIRIC, INSERM UMR 995, EA2686, Lille; m Dermatology Department, L’UNAM Université, University Hospital, Angers, France.

**Keywords:** acquired angioedema, acquired C1-inhibitor deficiency, monoclonal gammopathy, rituximab, splenic marginal zone lymphoma

## Abstract

Acquired angioedema (AAE) due to C1-inhibitor (C1INH) deficiency is rare. Treatment options for acute attacks are variable and used off-label. Successful treatment of the associated lymphoma with rituximab seems to prevent acute attacks in subjects with AAE. The aim of this study was to describe AAE manifestations, its associated diseases, and patients’ responses to treatments in a representative cohort.

A retrospective nationwide study was conducted in France. The inclusion criteria were recurrent angioedema attacks and an acquired decrease in functional C1INH <50% of the reference value.

A total of 92 cases were included, with a median age at onset of 62 years. Facial edema and abdominal pain were the most frequent symptoms. Fifteen patients were hospitalized in the intensive care unit because of laryngeal edema, and 1 patient died. Anti-C1INH antibodies were present in 43 patients. The associated diseases were primarily non-Hodgkin lymphoma (n = 44, with 24 splenic marginal zone lymphomas) and monoclonal gammopathy of undetermined significance (n = 24). Three patients had myeloma, 1 had amyloid light-chain (of immunoglobulin) (AL) amyloidosis, 1 patient had a bronchial adenocarcinoma, and 19 patients had no associated disease. Icatibant relieved the symptoms in all treated patients (n = 26), and plasma-derived C1INH concentrate in 19 of 21 treated patients. Six patients experienced thromboembolic events under tranexamic acid prophylaxis. Rituximab prevented angioedema in 27 of 34 patients as a monotherapy or in association with chemotherapy. Splenectomy controlled AAE in 7 patients treated for splenic marginal zone lymphoma. After a median follow-up of 4.2 years, angioedema was on remission in 52 patients.

AAE cases are primarily associated with indolent lymphoma—especially splenic marginal zone lymphoma—and monoclonal gammopathy of undetermined significance but not with autoimmune diseases or other conditions. Icatibant and plasma-derived C1INH concentrate control attacks; splenectomy and immunochemotherapy prevent angioedema in lymphoma setting.

## 1 Introduction

Bradykinin-mediated angioedema manifests as recurrent edema of the mucosa, soft tissue skin, and abdominal wall, usually during 2 to 5 days. This condition can be life threatening when the edema occurs in the tongue or laryngeal tract and causes asphyxia.^[1]^ The pathophysiological mechanisms result from the contact phase activation that is associated with kinin production caused by a deficiency in C1-inhibitor (C1INH), a serine protease inhibitor. Kallikrein (which induces bradykinin release from its precursor) and factor XII (which activates plasma kallikrein) are the primary targets for C1INH. In experimental studies, accumulation of kinins (primarily bradykinin) induces capillary vasodilatation via the activation of endothelial B1 and B2 receptors.^[2]^

Bradykinin-mediated angioedema can be hereditary or acquired. The hereditary angioedemas (HAEs) are usually caused by autosomal dominant inheritance of *SERPING1* gene mutations. *SERPING1* gene mutations result in either low C1INH expression (type I HAE) or normal levels with reduced C1INH function (type II HAE). HAE with normal C1INH levels and function is less frequent and is associated with *F12* gene mutations for 25% of the affected patients.^[3]^ Acquired angioedema with normal C1INH levels and function is related to the use of angiotensin-converting enzyme inhibitors.^[4]^

AAE that is associated with C1INH deficiency is rare, approximately 10 times more rare than the hereditary forms, which are estimated to occur in between 1/10,000 and 1/50,000 of the population.^[5]^ The largest series describing AAE represented <50 cases.^[6,7]^ A few clinical characteristics can help to distinguish AAE from HAE: in AAE, the disease typically develops after the fourth decade of life in patients with no familial history of angioedema, and with less frequent abdominal attacks. This condition is primarily associated with lymphoma and monoclonal gammopathy.^[6–8]^ Some cases are described with cancer or autoimmune conditions ^[7,8]^; in approximately 15% of cases, no associated condition was identified.^[7]^ A decrease of the functional C1INH level <50% of the reference value is commonly used to define the disease.^[7,9]^ Decreased levels of C4 and CH50 are regularly observed. C1q is also frequently decreased in AAE but is normal in HAE. A distinction between 2 subtypes has been suggested: one is characterized by C1INH consumption and is frequently associated with lymphoproliferative diseases, whereas the other is characterized by anti-C1INH antibodies and is thought to have an autoimmune mechanism.^[10]^ However, the relevance of this distinction is questioned, as AAE with anti-C1INH antibodies is also associated with monoclonal gammopathy and lymphoma.^[7,8]^

Treatments for angioedema attacks in AAE setting are used off-label. Plasma-derived C1INH concentrate (pdC1INH) efficiently treats attacks; however, some failures have been noted and suspected to be due to C1INH consumption.^[11]^ Icatibant, a competitive antagonist of the endothelial bradykinin B2 receptor, was reported to be effective in this setting in a small study.^[12]^ For prevention of angioedema attacks, patients with AAE exhibit a better response to antifibrinolytics than those with HAE,^[13,14]^ whereas the efficacy of attenuated androgens seems lower for this indication.^[7]^ Treatment of the underlying lymphoma with rituximab can prevent angioedema, particularly in AAE with anti-C1INH antibodies.^[15–22]^

We conducted this retrospective study to characterize AAE manifestations, to describe its associated diseases, and to observe the responses of angioedema to treatment.

## 2 Patients and methods

### 2.1 Design and setting

We conducted a retrospective study of AAE in France. All the procedures were performed in accordance with the principles expressed in the Declaration of Helsinki. Our institutional review board (Ile-de-France Committee no. 10) stated that all data collection and processing methods fulfilled these requirements. According to French legislation, no written informed consent of patients was required.

### 2.2 Participant inclusion and exclusion criteria

Our inclusion criteria were as follows: recurrent angioedema attacks, defined as cutaneous or mucosal edema that was resistant to antihistaminic or corticosteroid administration, first occurring after 40 years; and a decrease in functional C1INH <50% of the reference value, with decreased C1q and/or anti-C1INH antibodies. Patients with hereditary forms were excluded. Data concerning patients with asymptomatic decreases in functional C1INH were analyzed separately.

### 2.3 Data collection

Data collection extended from September 2013 to March 2015, and patients were referred through the immunology laboratories of Grenoble University Hospital and Georges Pompidou European Hospital in Paris, which are national reference laboratories for C1INH biology. We then contacted the clinicians to study the medical files.

First attack duration and attack frequency at first medical report were recorded. A cumulative record of different localizations of attacks presented over time by patients was established. If a disease was associated, the clinical, biological, and histological characteristics at diagnosis were recorded. AAE and its associated diseases were considered concomitant if the diagnosis delay was <4 months.

All samples were studied in immunology laboratory of Grenoble University Hospital or in immunology laboratory of Georges Pompidou European Hospital in Paris. The serum protein concentrations of C1INH, C4, and C1q were assayed by nephelometry (Siemens, Marburg, Germany). The complement hemolytic activity (CH50) was determined. Plasma C1INH function was assessed as the residual esterase activity in the plasma samples after incubation with the C1s protease. C1INH function was assayed as described by Drouet et al,^[23]^ or determined by chromogenic assay (Technochrom C1-inhibitor, Technoclone GmbH, Vienna, Austria). To quantify the presence of anti-C1INH autoantibodies, a slightly modified version of a C1INH-binding enzyme-linked immunosorbent assay was used.^[24]^ Isotype of C1INH antibodies was determined, but light chain component is not determined routinely.

### 2.4 Criteria of response to treatment

We considered the response of acute attacks of angioedema to *specific* treatment—tranexamic acid (TA), icatibant, or pdC1INH—as reported in medical files; we defined response as duration of an acute attack lasting <24 hours after administration of treatment. For *specific* preventive treatments—TA and danazol—and treatment of associated disease, we defined response as no attacks or a decrease of attack frequency by >50% during the 6 months following administration or introduction of treatment. The side effects data were also collected.

Disease status at the last available visit was assessed. Angioedema remission was defined as no attack in the previous 6 months. Biological remission was defined as a return to normal of C1INH function. The status of associated diseases, assessed by the clinician in the medical file, was recorded as complete remission or active disease—which means stability or progression.

### 2.5 Statistical analysis

We used StatView software (SAS Institute, Inc, Cary, NC, copyright 1992–1998). Median values were reported with the interquartile range (IQR) and mean values were reported with standard deviation. For group comparisons, we used Pearson χ^2^ test or analysis of variance test, if indicated. For remission survey assessments, we used the Kaplan–Meier and log-rank tests (Mantel–Cox). *P* values <0.05 were considered statistically significant.

## 3 Results

One hundred forty-four medical files were studied: 94 had been referred by the immunology laboratory of Grenoble University Hospital (where 540 patients with HAE are followed) and 50 by the immunology laboratory of Georges Pompidou European Hospital in Paris. Fifty-two patients were excluded: 1 because of *SERPING1* mutation, 33 because of lack of clinical information, and 18 patients who had asymptomatic C1INH decrease; however, biological characteristics and associated diseases of those asymptomatic patients were collected and are reported further separately. We thus included 92 patients; 56 were women (60%). The observational period extended from January 1991 to March 2015 with 4 patients diagnosed before 2000, 26 between 2000 and 2005, 29 between 2006 and 2010, and 33 between 2011 and 2015.

### 3.1 Characteristics of angioedema

The median age at first manifestation was 62 years (IQR = 18), and AAE diagnoses were made after a median delay of 10 months (IQR = 23).

**Figure 1:**
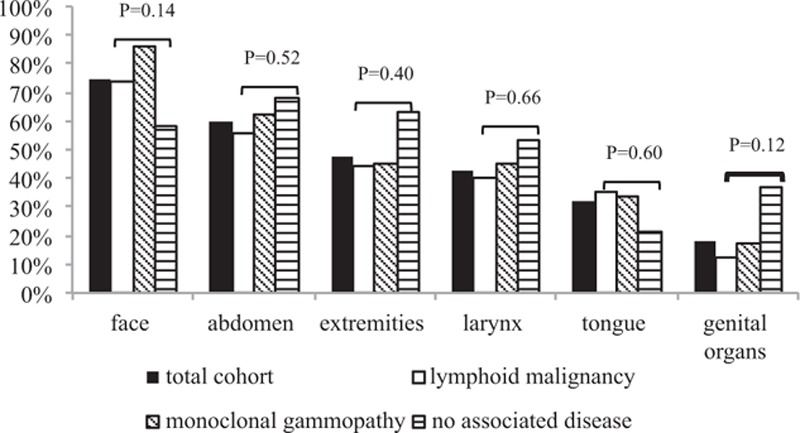
Cumulative record of localization of angioedema attacks over time among patients with AAE—general cohort and subgroups according to associated disease. A patient frequently has different localizations along time. χ^2^ test was applied for comparison between lymphoid malignancy, monoclonal gammopathy, and no associated disease subgroups. AAE = acquired angioedema.

The localizations of angioedema acute attacks are described in Fig. 1. The most frequent manifestations were facial angioedema (75% of total cohort) and abdominal pain (60%), followed by extremities (48%), larynx (43%), tongue (32%), and genital organs (18%). Three patients had only abdominal attacks without any peripheral angioedema. Fifteen patients were admitted into the intensive care unit because of asphyxia, and 1 patient was administered an unnecessary surgery because of an abdominal attack. Most of the patients (70%) experienced attacks lasting 24 to 72 hours; attacks lasted >72 hours in 18% of patients and <24 hours in 12% of patients. Thirty-nine percent of the patients had >1 attack per month.

The median biological values at diagnosis are presented in Table 1. C1q was low in all but 8 patients. All of the patients were tested for anti-C1INH antibodies, which were present in 43 patients (47%). Isotypes of anti-C1INH antibodies and associated disease are described in Table 2.

**Table 1:**
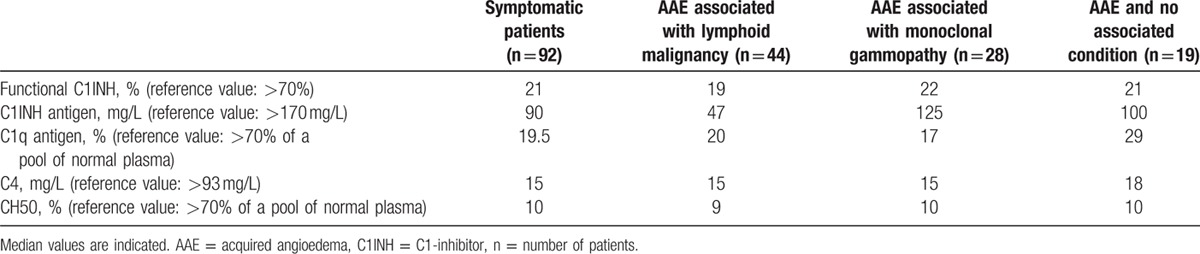
Biological characteristics of patients with acquired angioedema among general cohort and according to associated disease.

**Table 2:**
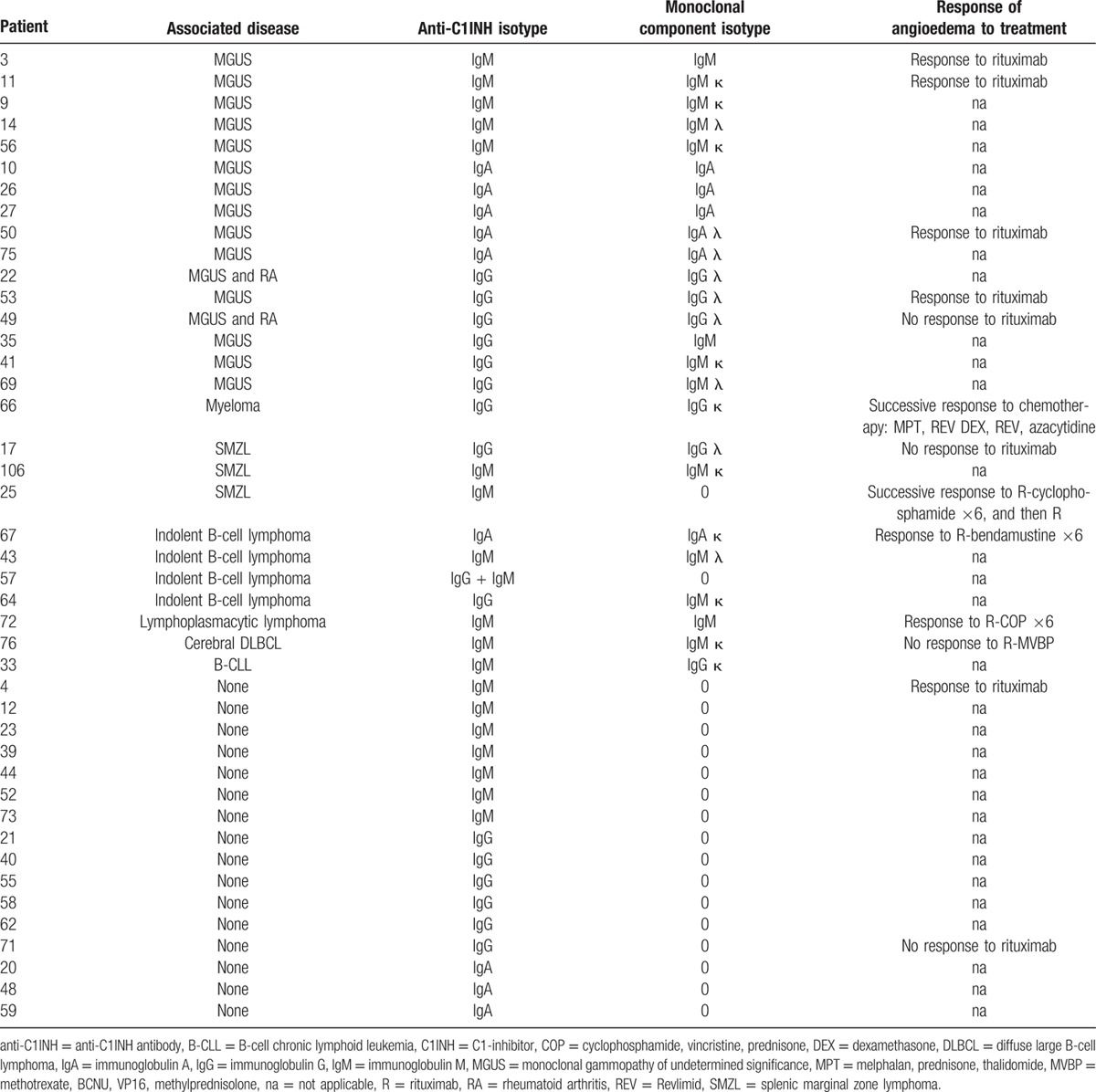
Isotypes of anti–C1-inhibitor antibodies and monoclonal component, and response to treatment, if administered.

We observed significant differences in sex, angioedema localization, and associated conditions according to the presence or absence of anti-C1INH antibodies (Table 3). AAE without anti-C1INH antibodies was more frequently observed in females, associated with lymphoid malignancy, and the C1q antigen levels were more frequently low. AAE with anti-C1INH antibodies was associated with more frequent attacks in the extremities, genital organs, and larynx, and more frequently associated with monoclonal gammopathy and idiopathic AAE. No statistical difference in clinical characteristics of angioedema was observed according to sex or presence of an associated disease (Fig. 1).

**Table 3:**
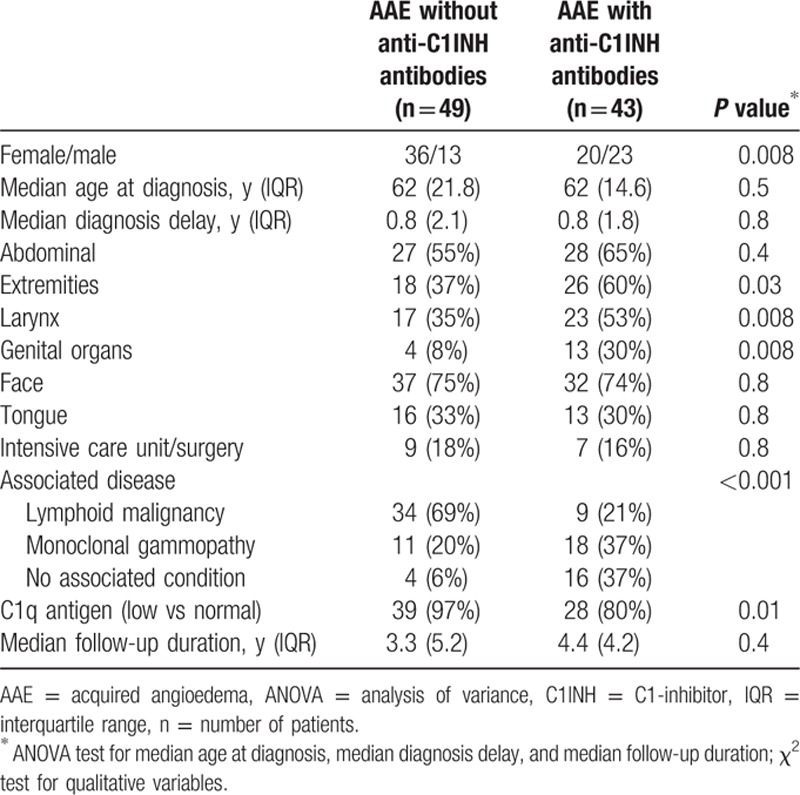
Characteristics of acquired angioedema according to presence or absence of anti–C1-inhibitor antibody.

### 3.2 Associated diseases

In 73 patients, an associated disease was identified (Table 4). No significant differences were observed in the occurrence or types of associated diseases according to date of diagnosis (data not shown).

**Table 4:**
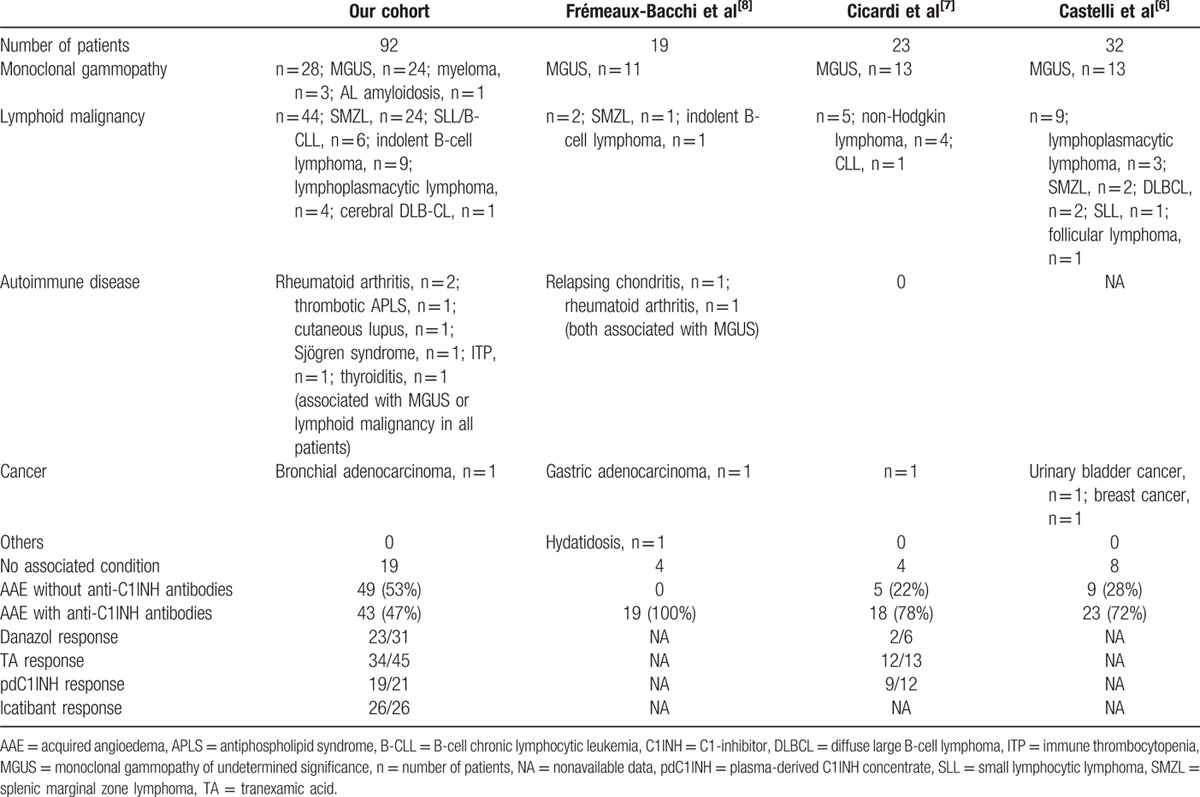
Description of our cohort and comparison with previous series of the literature of acquired angioedema with C1-inhibitor deficiency.

#### 3.2.1 Lymphoid malignancy

A lymphoid malignancy was diagnosed in 44 patients: splenic marginal zone lymphoma (SMZL) was diagnosed in 24 patients; indolent B-cell lymphoma, in 9 patients; small lymphocytic lymphoma/B-cell chronic lymphocytic leukemia, in 6 patients; lymphoplasmacytic lymphoma, in 4 patients; and cerebral diffuse large B-cell lymphoma, in 1 patient (Table 5 ). Among these 44 patients, 10 had anti-C1INH antibodies (Table 2).

**Table 5:**
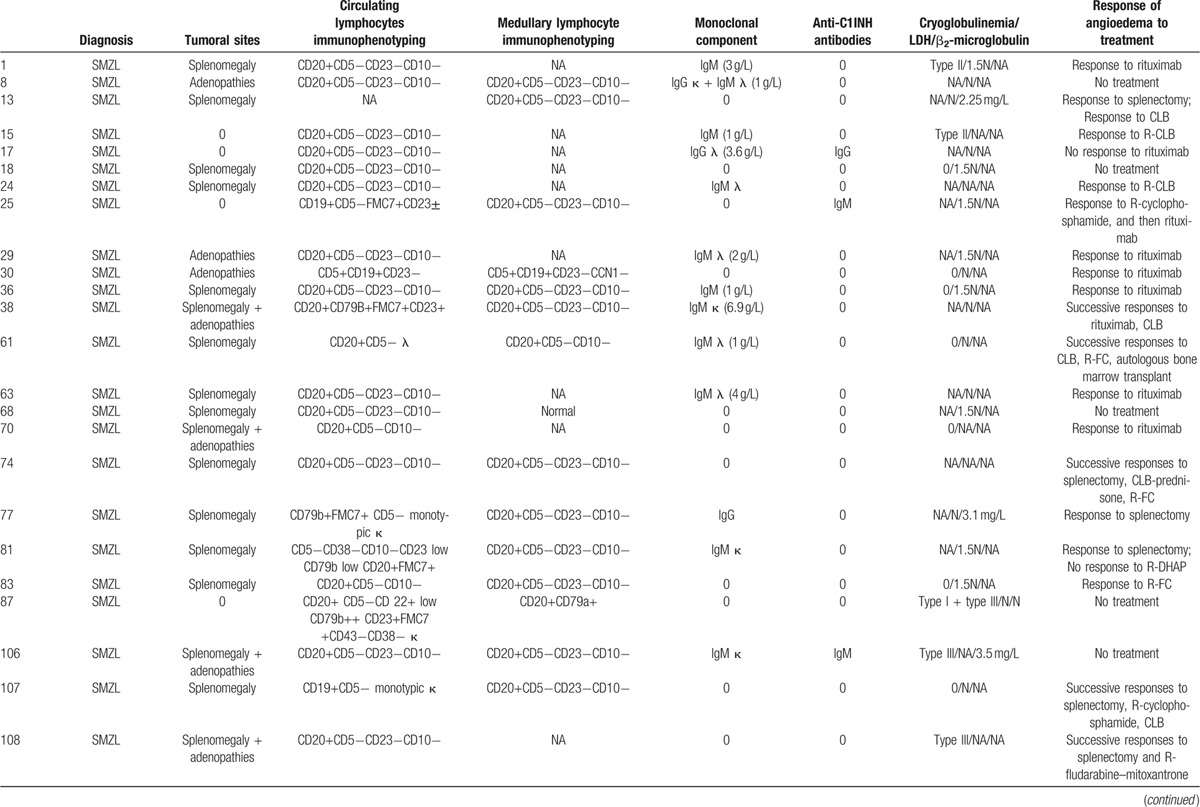
Clinical, immunological, and biological characteristics of lymphoid malignancies associated with acquired angioedema.

**Table 5 (Continued):**
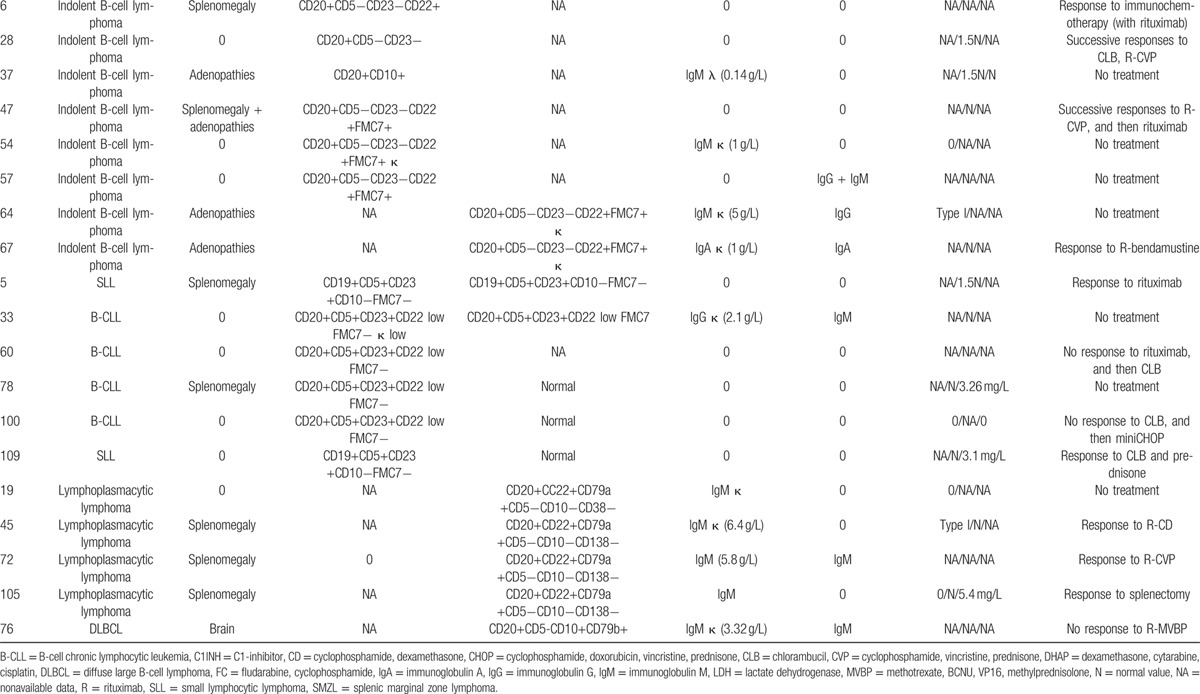
Clinical, immunological, and biological characteristics of lymphoid malignancies associated with acquired angioedema.

At diagnosis, the lactate dehydrogenase (LDH) value, available for 27 patients, was within the normal range in 16 cases and was 1.5 times above the reference value in 11 cases. The mean β_2_-microglobulin value was 2.29 mg/L (reference value <2.50 mg/L). Serologic tests for hepatitis C virus and human immunodeficiency virus were negative in all patients.

Lymphoid malignancy and angioedema diagnoses were concomitant in 25 patients, whereas lymphoid malignancy diagnosis preceded angioedema manifestations in 8 patients, with a median delay of 1.1 years (IQR = 2; range, 0.4–6.2), or followed angioedema in 11 patients, after a median delay of 1 year (IQR = 1.6; range, 0.4–5.0). The clinical and biological AAE characteristics in this setting were similar to the general pattern (Fig. 1; Table 1).

#### 3.2.2 Monoclonal gammopathy

A monoclonal gammopathy was associated with AAE in 28 patients, which included 24 monoclonal gammopathy of undetermined significance (MGUS), with a mean value of immunoglobulin 2.6 g/L (standard deviation, 1.0–13.0 g/L); 3 immunoglobulin G (IgG) myeloma cases; and 1 AL IgG amyloidosis case. Anti-C1INH antibodies were present in 17 patients (Table 2).

Monoclonal gammopathy and angioedema diagnoses were concomitant in 11 patients, whereas monoclonal gammopathy was diagnosed first in 6 patients, with a median delay of 1.0 year (IQR = 2.1; range 0.6–8.5), or after angioedema in 11 patients, after a median delay of 3.4 years (IQR = 2.5; range 0.6–8.5). The clinical and biological AAE characteristics in this setting were similar to the general pattern (Fig. 1; Table 1).

#### 3.2.3 Miscellaneous diseases

Seven patients, who displayed lymphoma or MGUS, also had autoimmune diseases: thrombotic antiphospholipid syndrome (n = 1), cutaneous lupus (n = 1), Sjögren syndrome (n = 1), immune thrombocytopenia (n = 1), Hashimoto thyroiditis (n = 1), or rheumatoid arthritis (n = 2). AAE without anti-C1INH antibodies was associated with bronchial adenocarcinoma in 1 patient.

#### 3.2.4 No associated disease

Nineteen patients never exhibited an associated condition, in spite of a median follow-up of 1.8 years (IQR = 4.4). Among these 19 patients, a total body computed tomography scan had been performed and was normal in 15, and a bone marrow biopsy had been performed and was normal in 4. Anti-C1INH antibodies were detected in 16 cases (Table 2). The clinical and biological AAE characteristics in this setting were similar to the general pattern, except C1q antigen value, higher than in the total cohort and other subgroups (Fig. 1; Table 1).

### 3.3 Treatment

#### 3.3.1 *Specific* treatment of angioedema

Response of angioedema to treatment of acute attacks and preventive treatment are detailed in Table 6.

**Table 6:**
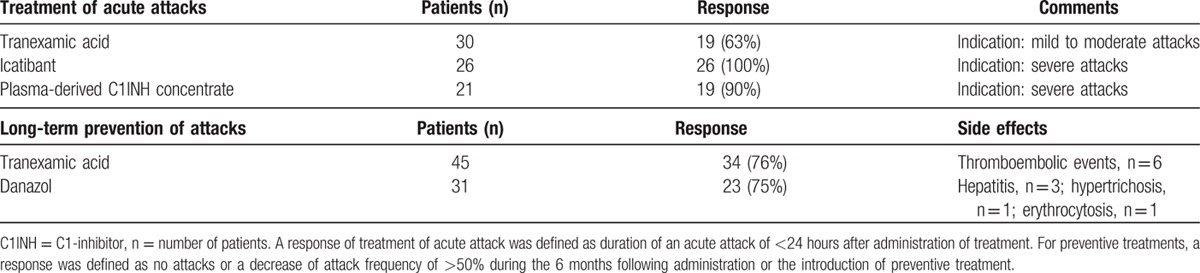
Response of angioedema to *specific* treatment.

A response to TA, which was used to treat moderate attacks, was observed in 63% of patients. pdC1INH, at standard dose of 20 UI/kg of body weight, was efficient in 90% of patients; a failure was observed in 2 patients, 1 of whom had anti-C1INH antibodies. Icatibant consistently reduced the attack durations in all 26 treated patients. No side effects were reported after those treatments.

TA and danazol were effective in preventing acute attacks, with a response occurring in 76% and 75% of the patients, respectively. The TA treatment was complicated by venous thromboembolic events in 6 patients. Among these patients, 3 displayed predisposing factors, which included antithrombin III deficiency, antiphospholipid syndrome, and a progressive diffuse large B-cell lymphoma. The danazol treatment was complicated in 5 patients.

#### 3.3.2 Associated-disease treatments and efficacy on angioedema

**Figure 2:**
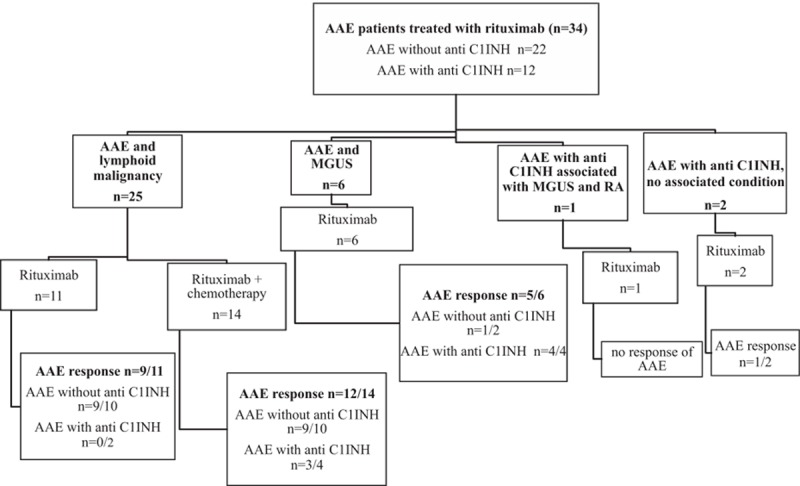
Rituximab administration and response of AAE. A response was defined as no attacks or a decrease of attack frequency of >50% during the 6 months following administration or introduction of rituximab. AAE = acquired angioedema, anti-C1INH = anti-C1INH antibody, C1INH = C1-inhibitor, MGUS = monoclonal gammopathy of undetermined significance, n = number of patients, RA = rheumatoid arthritis.

Rituximab was administered to 34 patients and was indicated for lymphoma (n = 10), frequent angioedema attacks (n = 14), or both (n = 10). The responses of AAE to rituximab, according to associated diseases and AAE subtypes, are described in Fig. 2. A response was observed in 21 of 25 patients with lymphoid malignancy, in 5 of 7 patients with MGUS, and in 1 of 2 patients with AAE with anti-C1INH antibodies, without associated disease. Overall, a response was observed in 27 of 34 patients (79%), including 19 of 22 (86%) patients with AAE without anti-C1INH antibodies and 8 of 12 (67%) patients with AAE with anti-C1INH antibodies. In the latter subgroup, 1 response to rituximab was observed among 4 subjects with IgG anti-C1INH antibodies AAE, 2 among 2 with immunoglobulin A anti-C1INH antibodies AAE, and 5 among 6 with immunoglobulin M anti-C1INH antibodies AAE (Table 2). Relapse of angioedema occurred in 9 cases, after a mean delay of 17 months.

Apart from the rituximab treatment, 4 lymphoproliferative disorders were treated with chemotherapy alone, which resulted in a response of AAE in 3 patients, and 7 patients with SMZL were treated with a splenectomy, with a response of AAE in all cases (Table 5 ). Myeloma was treated with chemotherapy in all 3 patients, and a response of AAE was observed in 2 patients; 1 patient was lost to follow-up. Cyclophosphamide and dexamethasone controlled the amyloidosis progression and reduced the AAE attack frequency. Treatment for rheumatoid arthritis with a combination of corticosteroids, hydroxychloroquine, and methotrexate prevented angioedema attacks in 1 patient, whereas methotrexate and then azathioprine prevented them in another patient. Hydroxychloroquine was used in 3 other patients and prevented angioedema attacks in 1 patient treated for a cutaneous lupus, and in 1 patient with AAE who displayed anti-C1INH antibodies, without any other disease.

### 3.4 Follow-up

Patients were followed for a median duration of 4.2 years (IQR = 6.8) after AAE diagnosis. Only 7 patients were lost to follow-up. Four patients died from the following: laryngeal angioedema revealing AAE with anti-C1INH antibodies (n = 1), lymphoma progression (n = 2), or intracerebral hemorrhage (n = 1).

**Figure 3:**
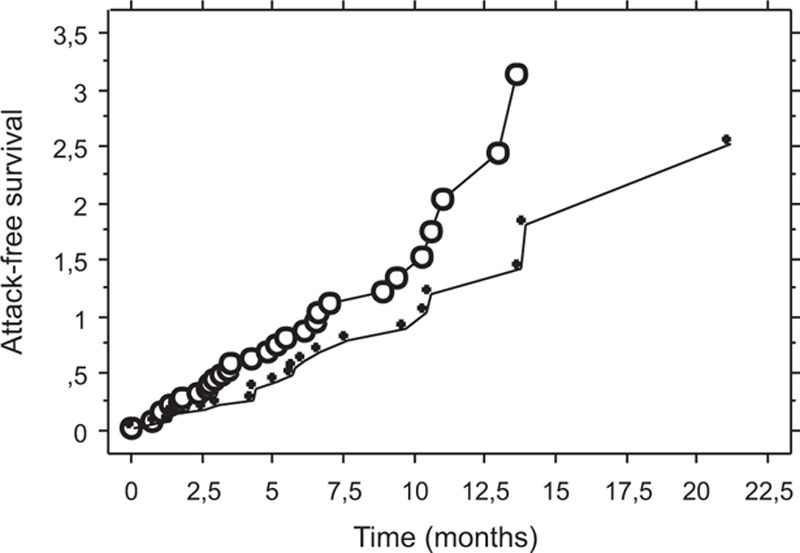
Attack-free survival among patients with acquired angioedema with anti-C1INH antibodies and patients with acquired angioedema without anti-C1INH antibodies. Kaplan–Meier analysis of survival without attacks among 41 patients with acquired angioedema without anti-C1INH antibodies (white circle line) and 40 patients with acquired angioedema with anti-C1INH antibodies (black circle line), for whom data were available; log-rank test *P* = 0.045. C1INH = C1-inhibitor.

At the last visit, angioedema was in remission in 52 patients: 31 of them were treatment-free and 21 patients received *specific* preventive treatment (TA or danazol). The associated disease, identified in 41 of those patients, was in complete remission in 28 patients and active in 13 patients. Twenty-nine patients had an active AAE disease at their last visit: 14 were under *specific* preventive treatment, 11 received icatibant or pdC1INH for occasional attacks, and 4 were treatment-free. The associated disease, identified in 22 of those patients, was in complete remission in 9 patients, and active in 13. No statistical link was found between the remission of associated conditions and AAE outcomes (data not shown). AAE without anti-C1INH antibodies was associated with a better attack-free survival than AAE with anti-C1INH antibodies (Fig. 3).

The biological data were examined at last visit for 54 patients, and a return to normal C1INH function was observed in only 8 patients, who all were in clinical remission. Among 46 patients with a persistent decrease in C1INH function at last visit, 25 had clinical remission of angioedema, including 12 treatment-free patients. Anti-C1INH antibodies were still present in 26 patients, 12 of whom had clinical remission of angioedema.

### 3.5 Characteristics of asymptomatic C1INH deficiency

Eighteen individuals with decreased C1INH function <50% of the reference value were asymptomatic, despite a median follow-up of 7.8 years (IQR = 8.7). In those patients, the decrease in C1INH was revealed by complement fraction C4 consumption in the absence of cryoglobulinemia; none of these patients displayed anti-C1INH antibodies. The median age at first observation was 61 years (IQR = 11.5). Incidental discovery was made in the settings of a lymphoma (n = 12), MGUS (n = 3), common variable immunodeficiency (n = 1), breast cancer (n = 1), and breast cancer associated with MGUS (n = 1). Their biological patterns were similar to the general cohort, except for slightly higher median C1q values: the mean value of functional C1INH was 30%; the mean value of C1INH antigen, 95 mg/L; the mean value of C1q antigen, 28.5%; the mean value of C4, 15 mg/L; and the mean value of CH50, 9%.

## 4 Discussion

This retrospective study describes the largest cohort of subjects with AAE due to C1INH deficiency. Our observations confirm previous results from smaller studies and allow for the description of new insights.

Although no national database for bradykinin angioedema exists in France, we could estimate the frequency of AAE as 11% of the 610 patients followed in Grenoble immunology laboratory, which confirms previous reports.^[7,9]^ AAE occurs primarily during the sixth decade of age, which clearly differentiates this condition from hereditary forms.^[5]^ Acute attacks of AAE are primarily localized to the face and abdomen. The latter can lead to a misdiagnosis; however, only 3 patients had isolated abdominal attacks, which likely explains the short diagnosis delay compared with HAE.^[5,25]^ Half of our patients experienced potentially life-threatening attacks because of a laryngeal or tongue localization, 1 had unnecessary surgery for abdominal attack, and 1 patient died of laryngeal attack, which confirms the need to diagnose and properly prevent attacks.

Our work allowed us also to identify 18 patients displaying an asymptomatic decrease in C1INH function, which was previously described in 6 patients with lymphoid disease.^[26,27]^ These cases were of asymptomatic C1INH deficiencies, and our description of clinical remission of angioedema occurrences without normalization of C1INH function raises some questions regarding the mechanisms that induce angioedema. Activation of the contact phase is crucial in angioedema attacks.^[28]^ Even if functional C1INH is very low, the activation of B1 and B2 receptors may be controlled. Angioedema severity depends on those endothelial receptor ligands that are essentially produced by the kinin-forming system. A recent assay was developed to evaluate C1INH function using contact phase proteases.^[29]^ The hydroxychloroquine efficacy in 3 of our patients, as described previously,^[30]^ supports the hypothesis of a high kinin-forming capacity in clinical AAE expression.

When comparing subtypes of AAE in our cohort, significant clinical differences were observed. AAE without anti-C1INH antibodies was associated with indolent lymphoid malignancy and with lower C1q values, suggesting that C1INH consumption occurred through C1 activation in the setting of high tumor mass lymphoid malignancies, despite low LDH and β_2_-microglobulin values. Concerning the immunological function of anti-C1INH antibodies, hypothesis can be raised, as inhibition of the enzymatic activity or enhancing the clearance of the molecule; however, to date, no functional tests are available in France.

We describe a striking association of AAE with SMZL, which was diagnosed in 24 patients in this cohort. The association of AAE with indolent B-cell lymphoma is well known; however, only a few cases of SMZL have been previously reported.^[6,8,15,18,31–35]^ A recent series describing lymphomas associated with AAE highlights this feature.^[36]^ SMZL is a rare lymphoid malignancy that affects patients in their sixth decade of age.^[37]^ This association seems significant because treatment for SMZL frequently induces an AAE response.

Autoimmune diseases were reported in only 7 patients in our cohort, and all of these patients displayed MGUS or lymphoid malignancy; only 1 patient had cancer. Reports regarding the association of autoimmune diseases and solid tumors with AAE are scarce,^[6,8,38,39]^ and might be fortuitous. Interestingly, we describe 3 cases of AAE associated with myeloma, which is quite rare in the literature.^[40]^

Among this cohort, B-cell lymphoproliferation appeared to be the primary associated feature of AAE, with or without anti-C1INH antibodies. However, the isotype of the monoclonal component and of the anti-C1INH antibodies did not strictly correlate.

Based on these results, we recommend that the diagnosis workup of AAE should include complete blood count, circulating lymphocyte immunophenotyping, LDH, plasmatic and urinary protein immunoelectrophoresis, and total body computed tomography scan. Bone marrow aspirate or osteomedullar biopsy should be performed in case of suspicion for lymphoma or myeloma, and in case of frequent attacks of angioedema. Annual screening should be repeated, especially if angioedema attacks are not prevented by a *specific* treatment.

Regarding the treatment response, despite a bias due to the retrospective nature of this study, our results allow us to reconsider therapeutic options, and to complete the recent consensus report.^[41]^ The response to TA for nonsevere attacks was moderate; in contrast, icatibant and pdC1INHs were very effective options for severe attacks in AAE. Ecallantide is not allowed for use in France, so we were not able to study its efficacy on AAE attacks. Prevention of attacks with antifibrinolytic agents and attenuated androgen was similar in our cohort, prompting us to recommend providing a preventive treatment to patients but avoiding antifibrinolytics if thromboembolic risk factors are present, since thromboembolic events occurred in 6 patients. Due to the potential severity of attacks, patients should be prescribed icatibant and C1INH concentrates in all cases and should be properly educated on autoinjections.

Treatment of associated diseases, especially the treatment of lymphoid malignancy, prevented acute attacks in most cases, regardless of the treatment options (splenectomy, rituximab, or immunochemotherapy). Efficacy of splenectomy had been reported in 1 case report.^[34]^ Rituximab had already been reported to reduce the frequency of angioedema attacks, particularly in the presence of anti-C1INH antibodies and in lymphoma settings.^[15–22,35]^ In our cohort, rituximab prevented angioedema attacks in 79% of the 34 treated patients, with a slightly better response in patients with lymphoid malignancies and no anti-C1INH antibodies. In patients with SMZL, splenectomy or anti-CD20 monotherapy—especially for patients who are reluctant to undergo surgery—is an interesting option for angioedema prevention. A response to rituximab was also observed in the MGUS-associated AAE, even 2 patients with immunoglobulin A and IgG monoclonal component, which is quite unexpected. These results support the role of B cells in the pathophysiological mechanisms that underlie AAE; anti-C1INH might thus be borne by a monoclonal component or be produced by polyclonal B cells, associated with a clonal lymphoproliferation.

We recommend a regular monitoring of C1INH function, complement parameters, and anti-C1INH antibodies under treatment, in order to better evaluate correlation between clinical and biological remission.

## 5 Conclusion

This study confirms the potential severity of AAE with C1INH deficiency, which must be properly managed to prevent life-threatening or disabling attacks. Considering the good response to icatibant and pdC1INH with few side effects, these treatments could be used in severe attacks. B-cell lymphoid malignancies (particularly SMZL) and MGUS are strongly associated with acquired C1INH deficiency. Treatment of the associated disease controls AAE manifestations. Rituximab could be proposed; however, we must determine the precise therapeutic scheme in prospective studies.

## References

[1] Kim SJ Brooks JC Sheikh J Angioedema deaths in the United States, 1979–2010. *Ann Allergy Asthma Immunol* 2014; 113:630–634. 25280464 10.1016/j.anai.2014.09.003

[2] Björkqvist J Sala-Cunill A Renné T Hereditary angioedema: a bradykinin-mediated swelling disorder. *Thromb Haemost* 2013; 109:368–374. 23306453 10.1160/TH12-08-0549

[3] Bork K Barnstedt SE Koch P Hereditary angioedema with normal C1-inhibitor activity in women. *Lancet* 2000; 356:213–217. 10963200 10.1016/S0140-6736(00)02483-1

[4] Zuraw BL Bernstein JA Lang DM A focused parameter update: hereditary angioedema, acquired C1 inhibitor deficiency, and angiotensin-converting enzyme inhibitor-associated angioedema. *J Allergy Clin Immunol* 2013; 131:1491–1493. 23726531 10.1016/j.jaci.2013.03.034

[5] Longhurst H Cicardi M Hereditary angio-oedema. *Lancet* 2012; 379:474–481. 22305226 10.1016/S0140-6736(11)60935-5

[6] Castelli R Deliliers DL Zingale LC Lymphoproliferative disease and acquired C1 inhibitor deficiency. *Haematologica* 2007; 92:716–718. 17488706 10.3324/haematol.10769

[7] Cicardi M Zingale LC Pappalardo E Autoantibodies and lymphoproliferative diseases in acquired C1-inhibitor deficiencies. *Medicine (Baltimore)* 2003; 82:274–281. 12861105 10.1097/01.md.0000085055.63483.09

[8] Frémeaux-Bacchi V Guinnepain M-T Cacoub P Prevalence of monoclonal gammopathy in patients presenting with acquired angioedema type 2. *Am J Med* 2002; 113:194–199. 12208377 10.1016/s0002-9343(02)01124-5

[9] Cicardi M Zanichelli A Acquired angioedema. *Allergy Asthma Clin Immunol* 2010; 6:14. 20667117 10.1186/1710-1492-6-14 PMC2925362

[10] Bouillet-Claveyrolas L Ponard D Drouet C Clinical and biological distinctions between type I and type II acquired angioedema. *Am J Med* 2003; 115:420–421. 14553889 10.1016/s0002-9343(03)00396-6

[11] Agostoni A Aygören-Pürsün E Binkley KE Hereditary and acquired angioedema: problems and progress: proceedings of the third C1 esterase inhibitor deficiency workshop and beyond. *J Allergy Clin Immunol* 2004; 114 (3 suppl):S51–S131. 15356535 10.1016/j.jaci.2004.06.047 PMC7119155

[12] Zanichelli A Bova M Coerezza A Icatibant treatment for acquired C1-inhibitor deficiency: a real-world observational study. *Allergy* 2012; 67:1074–1077. 22686628 10.1111/j.1398-9995.2012.02853.x

[13] Melamed J Alper CA Cicardi M The metabolism of C1 inhibitor and C1q in patients with acquired C1-inhibitor deficiency. *J Allergy Clin Immunol* 1986; 77:322–326. 3484761 10.1016/s0091-6749(86)80111-7

[14] Cugno M Cicardi M Agostoni A Activation of the contact system and fibrinolysis in autoimmune acquired angioedema: a rationale for prophylactic use of tranexamic acid. *J Allergy Clin Immunol* 1994; 93:870–876. 8182230 10.1016/0091-6749(94)90380-8

[15] Branellec A Bouillet L Javaud N Acquired C1-inhibitor deficiency: 7 patients treated with rituximab. *J Clin Immunol* 2012; 32:936–941. 22526593 10.1007/s10875-012-9691-2

[16] Ziakas PD Giannouli S Psimenou E Acquired angioedema: a new target for rituximab? *Haematologica* 2004; 89:ELT13. 15339702

[17] Levi M Hack CE van Oers MH Rituximab-induced elimination of acquired angioedema due to C1-inhibitor deficiency. *Am J Med* 2006; 119:e3–e5. 16887400 10.1016/j.amjmed.2005.09.018

[18] Lam DH Levy NB Nickerson JM Acquired angioedema and marginal zone lymphoma. *J Clin Oncol* 2012; 30:e151–e153. 22508826 10.1200/JCO.2011.38.9957

[19] Hassan A Amarger S Tridon A Acquired angioedema responding to rituximab. *Acta Derm Venereol* 2011; 91:733–734. 21691703 10.2340/00015555-1157

[20] Kaur R Williams AA Swift CB Rituximab therapy in a patient with low grade B-cell lymphoproliferative disease and concomitant acquired angioedema. *J Asthma Allergy* 2014; 7:165–167. 25506230 10.2147/JAA.S68548 PMC4259552

[21] Dreyfus DH Na CR Randolph CC Successful rituximab B lymphocyte depletion therapy for angioedema due to acquired C1 inhibitor protein deficiency: association with reduced C1 inhibitor protein autoantibody titers. *Isr Med Assoc J* 2014; 16:315–316. 24979840

[22] Bygum A Vestergaard H Acquired angioedema—occurrence, clinical features and associated disorders in a Danish nationwide patient cohort. *Int Arch Allergy Immunol* 2013; 162:149–155. 23921495 10.1159/000351452

[23] Drouet C Alibeu C Ponard D A sensitive method to assay blood complement C1-inhibitor activity. *Clin Chim Acta* 1988; 174:121–130. 3260154 10.1016/0009-8981(88)90379-8

[24] Alsenz J Bork K Loos M Autoantibody-mediated acquired deficiency of C1 inhibitor. *N Engl J Med* 1987; 316:1360–1366. 3494945 10.1056/NEJM198705283162202

[25] Banerji A Busse P Christiansen SC Current state of hereditary angioedema management: a patient survey. *Allergy Asthma Proc* 2015; 36:213–217. 25976438 10.2500/aap.2015.36.3824 PMC4405601

[26] Hory B Panouse-Perrin J Dupond JL Acquired C1 esterase inhibitor deficiency and lymphoproliferative syndromes. *Rev Med Interne* 1985; 6:266–271. 4048687 10.1016/s0248-8663(85)80116-8

[27] Grosbois B Goasguen J Leblay R Acquired deficiency of C1 esterase inhibitor and malignant lymphoproliferative syndromes. *Rev Med Interne* 1985; 6:464. 4070859 10.1016/s0248-8663(85)80109-0

[28] Björkqvist J de Maat S Lewandrowski U Defective glycosylation of coagulation factor XII underlies hereditary angioedema type III. *J Clin Invest* 2015; 125:3132–3146. 26193639 10.1172/JCI77139 PMC4563738

[29] Ghannam A Sellier P Defendi F C1 inhibitor function using contact-phase proteases as target: evaluation of an innovative assay. *Allergy* 2015; 70:1103–1111. 26010015 10.1111/all.12657

[30] Vincent D Ponard D Fiorella S Benefits of hydroxychloroquine in the treatment of a patient with angioedema due to acquired C1 inhibitor deficiency. *Ann Allergy Asthma Immunol* 2015; 114:68–70. 25457864 10.1016/j.anai.2014.10.008

[31] Jolles S Williams P Carne E A UK national audit of hereditary and acquired angioedema. *Clin Exp Immunol* 2014; 175:59–67. 23786259 10.1111/cei.12159 PMC3898555

[32] Phanish MK Owen A Parry DH Spontaneous regression of acquired C1 esterase inhibitor deficiency associated with splenic marginal zone lymphoma presenting with recurrent angio-oedema. *J Clin Pathol* 2002; 55:789–790. 12354812 10.1136/jcp.55.10.789 PMC1769784

[33] Gaur S Cooley J Aish L Lymphoma-associated paraneoplastic angioedema with normal C1-inhibitor activity: does danazol work? *Am J Hematol* 2004; 77:296–298. 15495244 10.1002/ajh.20195

[34] Lara-Jiménez MA Ruiz-Rivera L Magro-Checa C Acquired angioedema with C1 inhibitor deficiency secondary to splenic marginal zone B-cell lymphoma. *Rev Clin Esp* 2014; 214:e107–e109. 24970460 10.1016/j.rce.2014.05.014

[35] Ates O Sunar V Babacan T Acquired C1 esterase inhibitor deficiency in a marginal zone lymphoma patient treated with rituximab. *J BUON* 2015; 20:349. 25778339

[36] Castelli R Wu MA Arquati M High prevalence of splenic marginal zone lymphoma among patients with acquired C1 inhibitor deficiency. *Br J Haematol* 2016; 172:902–908. 26728240 10.1111/bjh.13908

[37] Matutes E Oscier D Montalban C Splenic marginal zone lymphoma proposals for a revision of diagnostic, staging and therapeutic criteria. *Leukemia* 2008; 22:487–495. 18094718 10.1038/sj.leu.2405068

[38] Gelfand JA Boss GR Conley CL Acquired C1 esterase inhibitor deficiency and angioedema: a review. *Medicine (Baltimore)* 1979; 58:321–328. 449665 10.1097/00005792-197907000-00004

[39] Széplaki G Varga L Szépvölgyi A Acquired angioedema associated with primary antiphospholipid syndrome in a patient with antithrombin III deficiency. *Int Arch Allergy Immunol* 2008; 146:164–168. 18204284 10.1159/000113521

[40] Martin-Garcia C Díez-Gómez ML Camacho E AAE and IgA myeloma. *Allergy* 2002; 57:965–966. 12269956 10.1034/j.1398-9995.2002.23832_11.x

[41] Cicardi M Aberer W Banerji A Classification, diagnosis, and approach to treatment for angioedema: consensus report from the Hereditary Angioedema International Working Group. *Allergy* 2014; 69:602–616. 24673465 10.1111/all.12380

